# Prognostic and Clinicopathological Role of PD-L1 in Endometrial Cancer: A Meta-Analysis

**DOI:** 10.3389/fonc.2020.00632

**Published:** 2020-04-30

**Authors:** Ling Lu, Yonghong Li, Rong Luo, Junhui Xu, Jie Feng, Mingqiang Wang

**Affiliations:** Department of Obstetrics and Gynecology, Wenjiang District People's Hospital of Chengdu, Chengdu, China

**Keywords:** PD-L1, endometrial cancer, prognosis, clinicopathological features, meta-analysis

## Abstract

**Background:** A series of studies have explored the prognostic value of programmed death-ligand 1 (PD-L1) in patients with endometrial cancer (EC); however, the results are controversial. Therefore, this meta-analysis was performed to estimate the associations between PD-L1 expression and the prognosis and clinicopathological features of EC.

**Methods:** A comprehensive literature search of PubMed, Web of Science, and Embase was conducted up until September 06, 2019. Pooled hazard ratios (HRs) and 95% confidence intervals (CIs) for overall survival (OS) and progression-free survival (PFS) were computed using the random-effects model (REM) or fixed-effects model (FEM). Odds ratios (ORs) and 95% CIs were calculated to evaluate the relationship between PD-L1 and clinicopathological factors.

**Results:** A total of 9 studies with 1,615 patients were included in the meta-analysis. The combined data showed that high expression of PD-L1 was not significantly correlated with OS (HR = 1.20, 95% CI = 0.41–3.52, *p* = 0.737) or PFS (HR = 1.12, 95% CI = 0.50–2.54, *p* = 0.778) in EC. In addition, PD-L1 expression was significantly associated with poor differentiation (OR = 2.82, 95% CI = 1.96–4.06, *P* < 0.001) and advanced stage (OR = 1.71, 95% CI = 1.12–2.60, *p* = 0.013).

**Conclusion:** This meta-analysis suggests that PD-L1 expression is not associated with poor prognosis in patients with EC. However, PD-L1 expression is positively correlated with poor differentiation and advanced tumor stage in EC.

## Introduction

Endometrial cancer (EC) is the most common malignancy of the female genital tract in developed countries (1). There were nearly 100,000 new cases of EC in Europe in 2012, and the age-standardized incidence is 13.6/100,000 women (2). More than 90% of EC cases occur in patients aged >50 years, whereas 4% of new cases are younger than 40 years old (2). The majority of EC cases are diagnosed in stage I, with a 5-year survival rate of 95%. However, the 5-year survival rates dramatically decrease to 68 and 17% in regional spread or distant metastatic disease (2). Prognostic assessment is pivotal for clinical management and determining treatment regimens. Although previous evidence shows that several factors, including obesity, diabetes, and insulin resistance, are associated with inferior prognosis in EC, the survival prediction of individual patients is still difficult and challenging (1). Therefore, more accurate and predictive markers are urgently needed to monitor the disease progression of EC.

Accumulating evidence has revealed that cancer cells can use many important mechanisms to evade immune surveillance and to progress and metastasize (3). The programmed death-ligand 1 (PD-L1)/programmed cell death-1 (PD-1) axis is an immunosuppressive pathway that facilitates immune escape of tumor cells (4). PD-L1 expression has been shown to be a prognostic marker of prognosis in various cancers, including lung cancer, esophageal squamous cell carcinoma, pancreatic cancer, and colorectal cancer (5–8). Many studies have also explored the prognostic value of PD-L1 in EC, with conflicting results (9–17). Some investigators have reported that elevated PD-L1 expression is associated with shorter survival (16). However, some researchers showed that the predictive value of PD-L1 expression was not significant (17). Yamashita et al. reported that high PD-L1 expression was correlated with superior progression-free survival (*p* = 0.033) (14). Therefore, we utilized the existing literature to meta-analyze several independent studies to quantitatively clarify the prognostic role of PD-L1 in patients with EC.

## Materials and Methods

### Search Strategy

All procedures mentioned below were implemented in accordance with the Preferred Reporting Items for Systematic Reviews and Meta-Analyses statement (18). As this study analyzed data from previously published publications, ethical approval and patient consent were not required. A comprehensive literature search of PubMed, Web of Science, and Embase was performed with the following keywords: (“PD-L1” OR “programmed death ligand 1” OR “programmed cell death ligand 1” OR “B7-H1” OR “CD274”) AND (“endometrial cancer” OR “endometrial carcinoma” OR “endometrium cancer” OR “endometrial neoplasms”). The last search was conducted on September 06, 2019. The reference lists of relevant publications were also manually checked for possible eligible studies.

### Inclusion and Exclusion Criteria

Eligible studies must meet the following inclusion criteria: (1) the patients were histologically confirmed to be diagnosed with EC; (2) PD-L1 protein expression was evaluated in primary cancer tissues using immunohistochemistry (IHC); (3) studies evaluating the association between PD-L1 and survival outcomes and/or clinicopathological features. If the hazard ratio (HR) and 95% confidence interval (CI) were not directly reported in the text, sufficient data should be provided for calculation using Tierney's method (4, 19) studies with adequate sample size (n>20); (5) articles written in English. The exclusion criteria were as follows: (1) studies that were reviews, conference abstracts, case reports, comments, or bioinformatical analysis; (2) studies lacking necessary information.

### Data Extraction and Quality Evaluation

Two investigators (LL and YL) independently reviewed and extracted information from eligible studies, and all disagreements were settled by discussion with a third investigator (RL). The following data were extracted from the included articles: first author, year of publication, country, sample size, age, study period, detection method, tumor type, FIGO stage, study design, HR and 95% CI of overall survival (OS) and progression-free survival (PFS), and clinicopathological factors. The quality assessment of eligible studies was conducted according to the Newcastle–Ottawa scale (NOS) (20). The NOS contains 9 stars divided into 3 categories: selection (4 stars), comparability (2 stars), and outcome (3 stars). An NOS score of 0 to 9 was used to indicate the quality of the studies, and studies with NOS ≥6 were considered high quality.

### Statistical Analysis

Pooled HRs and 95% CIs were used to evaluate the association between PD-L1, OS, and PFS. An HR>1 without a 95% CI containing 1 indicates that PD-L1 overexpression predicts lower survival. The statistical heterogeneity of the eligible studies was estimated using the Cochran Q test and Higgins I-squared (*I*^2^) statistic. *I*^2^> 50% and P for Q test <0.10 indicate significant heterogeneity, and the random-effects model (REM) was applied to analyze pooled HRs and 95% CIs. Otherwise, a fixed-effects model (FEM) was adopted for the calculation. Subgroup analysis was conducted on the basis of sample size and geographic region. The relationship between PD-L1 expression and clinicopathological features was evaluated using combined odds ratios (ORs) and 95% CIs. Begg's test was used to detect publication bias. All statistical analyses were performed using Stata 12.0 (Stata Corporation, College Station, USA). *P* < 0.05 was considered statistically significant.

## Results

### Selection of Studies

**Figure 1:**
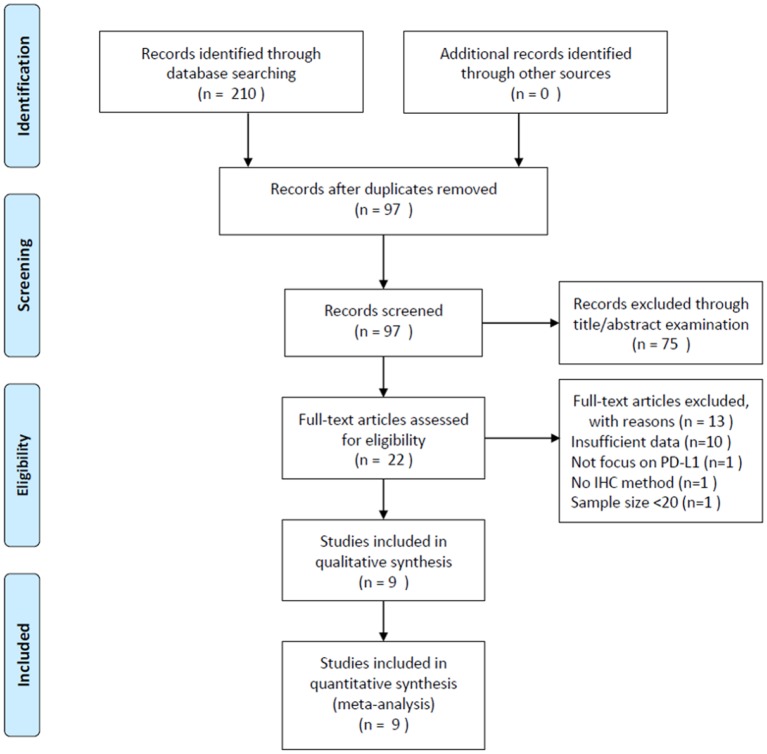
Literature review process.

A total of 210 studies were identified through a literature search. After duplicate records were removed, 97 studies were screened using their titles and abstracts. Of these studies, 75 records were excluded and 22 studies were selected for full-text examination. Subsequently, 13 articles were discarded for the following reasons: 10 studies did not provide sufficient data, one study did not focus on PD-L1, one study did not employ the IHC method, and one study had a sample size <20. Finally, 9 studies (9–17) with 1,615 patients were included in the meta-analysis. The literature selection process is illustrated in Figure 1.

### Characteristics of Included Studies

The included studies were published from 2016 to 2019, indicating the most recent attention on this issue (Table 1). The total sample size was 1,615, with individual sample sizes ranging from 53 to 700. The included studies were from 7 countries, including 2 studies in the USA (11, 15), 2 studies in Turkey (12, 16), and one each in China (9), Korea (10), Egypt (13), Japan (14), and Greece (17). All eligible IHC methods were used to detect PD-L1 expression in cancer tissue and a retrospective study design was used. Eight studies enrolled patients with FIGO stages I–IV (10–17) and one study recruited patients with FIGO stages I–III (9). Four studies (10, 14, 16, 17) provided data on the association between PD-L1 and OS, and four other studies (10, 11, 14, 17) reported information on PFS. Seven studies (9–13, 15, 17) investigated the relationship between PD-L1 and clinical factors. The NOS scores ranged from 6 to 9, indicating the high methodological quality of the included studies.

**Table 1:** Baseline characteristics of included studies.

**References**	**Country**	**Sample size**	**Mean age**	**Study duration**	**Detection method**	**Tumor type**	**FIGO stage**	**Study design**	**NOS score**
Mo et al. (9)	China	75	57.3	2012–2014	IHC	Mixed	I-III	Retrospective	7
Kim et al. (10)	Korea	183	53	2007–2017	IHC	Mixed	I-IV	Retrospective	8
Li et al. (11)	USA	700	60.5	2012–2015	IHC	Mixed	I-IV	Retrospective	7
Sungu et al. (12)	Turkey	127	62.9	2006–2016	IHC	Mixed	I-IV	Retrospective	9
Tawadros et al. (13)	Egypt	95	54.6	2008–2014	IHC	Mixed	I-IV	Retrospective	7
Yamashita et al. (14)	Japan	149	NR	2006–2017	IHC	Mixed	I-IV	Retrospective	6
Crumley et al. (15)	USA	132	60	2013–2016	IHC	Mixed	I-IV	Retrospective	7
Gulec et al. (16)	Turkey	53	61.8	1996–2015	IHC	Mixed	I-IV	Retrospective	8
Vagios et al. (17)	Greece	101	64.4	2001–2017	IHC	Mixed	I-IV	Retrospective	8

*IHC, immunohistochemistry; NOS, Newcastle-Ottawa scale; NR, not reported*.

### PD-L1 and Prognosis

**Figure 2:**
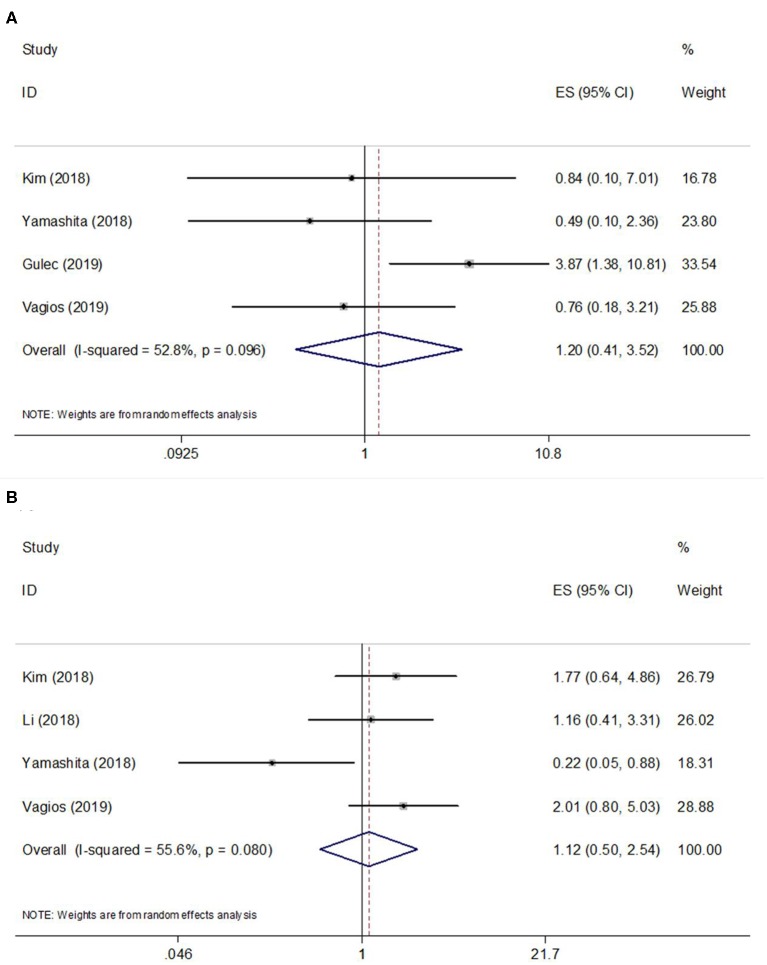
Forest plots of studies evaluating the hazard ratios (HRs) and 95% confidence intervals (CIs) of high PD-L1 expression in EC. **(A)** Forest plots of overall survival (OS); **(B)** Forest plots of progression-free survival (PFS).

A total of 4 studies (10, 14, 16, 17) provided data on PD-L1 expression and OS, and the heterogeneity was statistically significant (*I*^2^ = 52.8%, *p* = 0.098) (Table 2; Figure 2A); therefore, a REM was used. The pooled data were as follows: HR = 1.20, 95% CI = 0.41–3.52, *p* = 0.737, indicating that PD-L1 overexpression had a non-significant association with OS. Subgroup analysis of OS demonstrated that PD-L1 did not predict OS irrespective of sample size or geographic region (Table 2). Regarding PFS, 4 included studies (10, 11, 14, 17) presented relevant data. The combined results were HR = 1.12, 95% CI = 0.50–2.54, and *p* = 0.778 (Table 2; Figure 2B). Subgroup analysis showed that PD-L1 expression was not a significant factor for PFS regardless of sample size or geographic region (Table 2).

**Table 2:** Subgroup analysis of PD-L1 and OS and PFS in EC.

**Subgroup**	**No. of studies**	**OR (95%CI)**	***p***	**Effects model**	**Heterogeneity**	
					***I*^**2**^(%)**	**P**
Overall survival						
Total	4	1.20 (0.41–3.52)	0.737	REM	52.8	0.098
Sample size						
≤ 120	2	1.86 (0.38–9.09)	0.443	REM	69.2	0.071
>120	2	0.59 (0.17–2.10)	0.418	FEM	0	0.687
Geographic region						
Asian	3	1.35 (0.32–5.61)	0.682	REM	61.9	0.072
Non-Asian	1	0.76 (0.18–3.21)	0.709	–	–	–
Progression-free survival						
Total	2	1.12 (0.50–2.54)	0.778	REM	55.6	0.080
Sample size						
≤ 120	1	2.01 (0.80–5.04)	0.137	–	–	–
>120	3	0.86 (0.29–2.57)	0.794	REM	62.2	0.071
Geographic region						
Asian	2	0.67 (0.09–5.11)	0.702	REM	80.6	0.023
Non-Asian	2	1.58 (0.79–3.16)	0.193	FEM	0	0.440

### PD-L1 and Clinicopathological Features

**Figure 3:**
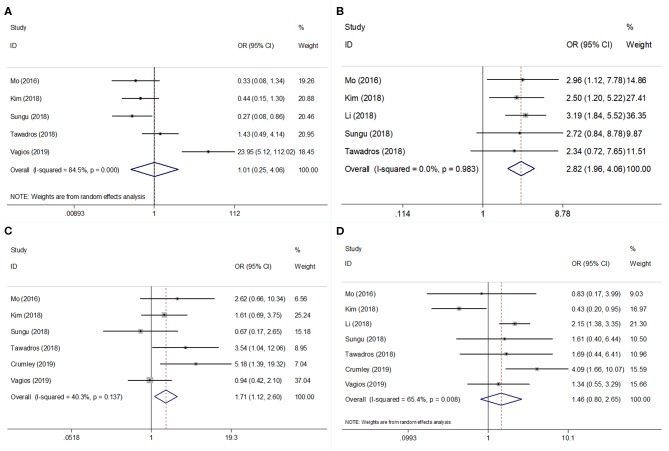
Forest plots for PD-L1 expression and clinicopathological features in EC. Meta-analysis of association between PD-L1 and **(A)** histological type; **(B)** differentiation; **(C)** tumor stage; and **(D)** lymphovascular space invasion (LVSI) in EC.

Seven studies evaluated the correlation between PD-L1 and clinicopathological factors, including histological type, differentiation, stage, and lymphovascular space invasion (LVSI)(9–13, 15, 17). The pooled ORs and 95% CIs showed that elevated PD-L1 expression was correlated with poor differentiation (OR = 2.82, 95% CI = 1.96–4.06, *P* < 0.001) and advanced stage (OR = 1.71, 95% CI = 1.12–2.60, *p* = 0.013) (Figure 3, Table 3). However, there was no significant relationship between PD-L1 and histological type (OR = 1.01, 95% CI = 0.25–4.06, *p* = 0.987) or LVSI (OR = 1.46, 95% CI = 0.80–2.65, *p* = 0.218).

**Table 3:** Correlations between PD-L1 expression and clinical characteristics in endometrial cancer.

**Clinicopathological features**	**No. of studies**	**OR (95%CI)**	***p***	**Effects model**	**Heterogeneity**	
					***I*^**2**^**	**(%) P**
Histological type (Endometrioid vs. Non-endometrioid)	5	1.01 (0.25–4.06)	0.987	REM	84.5	<0.001
Differentiation (Poor vs. Moderate/well)	5	2.82 (1.96–4.06)	<0.001	FEM	0	0.983
Stage (III-IV vs. I-II)	6	1.71 (1.12–2.60)	0.013	FEM	40.3	0.137
LVSI (Yes vs. No)	7	1.46 (0.80–2.65)	0.218	REM	65.4	0.008

*LVSI, lymphovascular space invasion; OR, odds ratio*.

### Publication Bias

**Figure 4:**
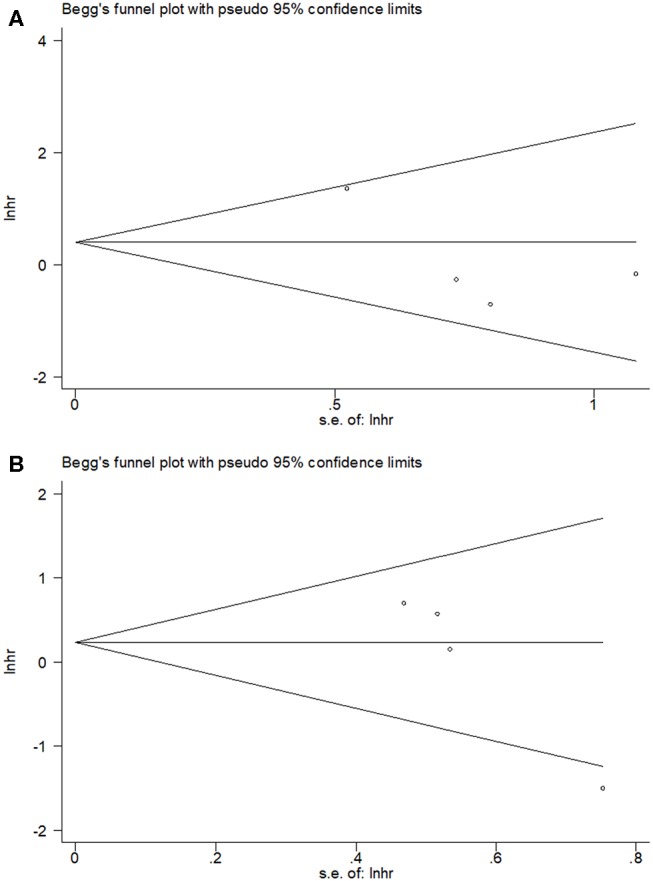
Begg's funnel plot of PD-L1 for **(A)** OS and **(B)** PFS.

By using Begg's test, we estimated the publication bias of the included studies regarding OS and PFS. As shown in Figure 4, there was no clear evidence of funnel plot asymmetry by visual assessment. The Begg's *p*-values were *p* = 0.734 for OS and *p* = 0.089 for PFS, which suggested that there was no significant publication bias in this meta-analysis.

## Discussion

The prognostic value of PD-L1 in EC has been explored in previous studies (9–17), but the results were conflicting. We systemically searched eligible studies covering this topic and meta-analyzed the data. The pooled results showed that PD-L1 overexpression had a non-significant impact on OS or PFS in patients with EC. However, high PD-L1 expression was associated with poor differentiation and advanced stage, suggesting that PD-L1 may be involved in the aggressive biological behavior and metastasis of EC cells. Our results provide evidence that PD-L1 overexpression could be a potentially predictive marker of poor differentiation and distant or local metastasis of EC. To the best of our knowledge, the current study is the first meta-analysis investigating the prognostic value of PD-L1 for EC. The results may provide important implications for EC treatment.

In the tumor microenvironment, PD-L1 overexpression can generate immunosuppressive effects, and the engagement of PD-1/PD-L1 can prompt the temporary downregulation of T cell function, tumor-associated macrophages (TAMs), and NK cells in various cancers (21–23). Growing evidence shows that the PD-1/PD-L1 signaling pathway is involved in tumor immune escape and therefore promotes cancer cell survival (24). Immune checkpoint inhibitors (ICIs) blocking the PD-1 receptor and its ligand PD-L1 have revolutionary therapeutic effects in cancer patients (25). Tumor-intrinsic PD-L1 plays an important role in facilitating cancer initiation, stemness formation, and tumor invasion (26). ICIs have shown a favorable safety profile and potent antitumor activity in PD-L1 positive EC patients following chemotherapy (27). A recent multicenter, single-arm, phase 2 trial showed that lenvatinib plus pembrolizumab showed anti-tumor activity in patients with advanced recurrent EC with a safety profile (28).

The prognostic value of PD-L1 was also investigated in previous studies using a meta-analytic approach. A meta-analysis including 11 studies demonstrated that PD-L1 overexpression was a predictor of worse survival outcomes in bladder cancer (29). Furthermore, high PD-L1 was significantly correlated with later tumor stages (OR = 3.9, 95%CI = 2.71.5.61, *p* < 0.001) and distant metastases (OR = 2.5, 95%CI = 1.22.5.1, *p* = 0.012) in bladder cancer (29). Another meta-analysis on non-small cell lung cancer (NSCLC) showed that PD-L1 expression was significantly associated with histology type, differentiation, and tumor stage, whereas the relationship between PD-L1 expression and OS was not statistically significant (*p* = 0.863) (30). Similar to this study on NSCLC (30), we also found an association between PD-L1 and differentiation and tumor stage in EC, whereas the prognostic effect of PD-L1 on survival was not significant. A meta-analysis conducted by Italian researchers also suggested that high PD-L1 expression does not correlate with poor prognosis of patients with oral squamous cell carcinoma (31). Our finding that PD-L1 does not predict survival in EC may be due to the limited sample size of the included studies. After all, data from only 4 studies was aggregated for the analysis of OS and PFS, and this relatively small sample size could possibly restrain the significant prognostic impact of PD-L1 on outcomes. Therefore, more large-scale cohort studies on PD-L1 and EC are necessary for validation that is more accurate.

Although we made an effort to perform a comprehensive meta-analysis, several limitations should still be taken into account when interpreting our results. First, the sample size was relatively small, which may have introduced selection bias. Second, eligible studies used different antibodies and cut-off values to determine PD-L1 positivity. These variances in multiple methodologies could lead to potential heterogeneity. Third, the data extracted from eligible studies were the HR and 95% CI of the patient group, rather than individual patient data. Therefore, the accuracy of the pooled results may be compromised.

## Conclusions

In summary, with the aim of estimating the prognostic efficiency of PD-L1 expression in EC, this study provides an intensive overview of survival outcomes and clinical factors. According to our results, PD-L1 expression was not associated with poor prognosis in patients with EC. However, PD-L1 expression was positively correlated with poor differentiation and advanced tumor stage.

## Data Availability Statement

All datasets generated for this study are included in the article/supplementary files.

## Author Contributions

LL and YL designed the project. LL, YL, and RL performed data extraction and analysis. JX and JF performed the quality assessment. JX, JF, and MW contributed to the article drafting. LL and YL revised the manuscript critically and supervised the project. All authors read and approved the final manuscript.

## Conflict of Interest

The authors declare that the research was conducted in the absence of any commercial or financial relationships that could be construed as a potential conflict of interest.
